# Influence of Photoinitiator Type and Curing Conditions on the Photocuring of Soft Polymer Network

**DOI:** 10.3390/ma16237348

**Published:** 2023-11-25

**Authors:** Malwina J. Niedźwiedź, Gokhan Demirci, Nina Kantor-Malujdy, Miroslawa El Fray

**Affiliations:** Department of Polymer and Biomaterials Science, Faculty of Chemical Technology and Engineering, West Pomeranian University of Technology in Szczecin, Al. Piastów 45, 70-311 Szczecin, Poland

**Keywords:** polymer networks, photo-DSC, photocuring, fatty acid, polyester diol, telechelic macromonomer, methacrylates, renewable resources

## Abstract

The presented work deals with the photocuring of telechelic macromonomers derived from plant-based fatty acids to obtain a soft polymer network. Compositions were made by mixing macromonomers with three different concentrations (0.5, 1, and 2%) of two type I photoinitiators (Omnirad 2022 and Omnirad 819). All formulations were then subjected to photopolymerization studies by applying UV-assisted differential scanning calorimetry (UV-DSC) measurements at isothermal conditions at 37 °C with a narrow light source wavelength of 365 nm and irradiation (light intensity) of 20 and 50 mW/cm^2^. The percentage conversions, reaction orders, and constants were estimated based on autocatalytic Sestak–Berggen and Avrami models. In this work, for the first time, the influence of the curing conditions on the photopolymerization process, such as the photoinitiator concentration, light intensity, and oxygen presence/absence, were investigated for these novel systems. The results indicated significant differences between the two commercially available photoinitiators and their effects on photopolymerization kinetics. The maximum reaction rate was found to be considerably higher for Omnirad 2022 (which is a blend of three different compounds), especially at a lower light intensity, i.e., 20 mW/cm^2^, compared to Omnirad 819. However, it led to lower maximum conversion in an air atmosphere. The dynamic thermomechanical analysis (DMTA) revealed that light intensity, photoinitiator concentration, and oxygen presence had a strong effect on the storage modulus and loss modulus values. It was concluded that the chemical structure of the photoinitiator and curing conditions had a strong effect on the photopolymerization kinetics and properties of the prepared soft polymer networks.

## 1. Introduction

Photopolymerization is one of the frequently used methods for the manufacturing of polymeric materials, including thin films utilized in the coating and adhesive industries, as well as in advanced high-tech applications [1,2]. UV curing is a highly advantageous method due to its low energy consumption, rapid curing time (often in seconds), and low reaction temperature. When non-toxic and “green” monomers/precursors are utilized in photopolymerization, the whole process becomes sustainable and environmentally friendly.

Nowadays, coating materials based mainly on polyurethanes, especially those based on bio-polyols, are attracting increasing attention [3]. They can be obtained by many different methods; however, photopolymerization is attracting the greatest interest, especially in the fields of medical adhesives and tissue engineering [4] and drug-delivery systems [5,6]. T. Yuan et al. used biomass polyols to synthesize a multifunctional polyurethane acrylate that significantly improved the green performance of UV-curable coatings. Adjusting the contents of the different biomass components resulted in the shift of the polymer’s thermal stability. Moreover, only 30 s was sufficient to obtain UV-cured polymers [3]. X. He and colleagues synthesized a trifunctional itaconic acid-based crosslinking agent (IHA) for use in the production of green UV-curable waterborne polyurethane (WPU) coatings, where the entire process was conducted at temperatures below 100 °C [7]. Due to its numerous advantages, photocuring is the primary method for obtaining dental resins [8,9]. Moreover, it is widely used in high-tech applications, such as micromachining [10], 3D printing [11,12,13,14,15], optoelectronics [16], and solvent-free paint.

Besides its many advantages, photopolymerization also has some limitations [17]. The depth of light penetration through the material and oxygen inhibition are the limiting factors for possible applications [18]. The oxygen inhibition phenomenon can occur in certain chemical reactions, such as the photocuring process in polyurethane systems. It can limit the diffusion of oxygen, leading to a reduction in the rate of the reaction and, potentially, the incomplete curing of the material. To minimize its effects, additives or other techniques can be used to stop the promotion of oxygen diffusion [19]. Therefore, photopolymerization is used for thin-film preparation, mainly in the coating [20,21] and adhesive [22] industries and advanced high-technology applications [23,24,25,26,27,28].

The structure of the monomer is the most important factor influencing the kinetics of photocrosslinking and, thus, the final properties of the resulting network [29]. The presence of urethane groups significantly accelerates the photopolymerization process and leads to higher conversion. This can be attributed to the pre-association of the molecules through strong hydrogen bonding. Moreover, hydrogen bonds increase the viscosity, which limits oxygen diffusion (reduces oxygen inhibition), allowing for greater conversion [30].

The length of the monomer mainly influences segmental diffusion, which is crucial in order to achieve the highest maximum conversion. For example, the hardness, curing rate, and conversion in the photopolymerization of polyurethanes increase with the decrease in the polyol’s molar mass. This is mainly because compositions based on shorter-chain polyols have greater reactivity, and a higher relative concentration of methacrylate double bonds in the system forms stronger polymer networks [31].

The inherent components of the photocuring system are the photoinitiator and its concentration. Generally, two types of photoinitiators can be distinguished based on the path of the generation of free radicals after reaching an excited state [32]. The rate of initiation and the generation of more radicals at the beginning of the reaction are accelerated by a higher initiator concentration, which extends the period during which the system retains mobility. In addition, the rapid increase in viscosity helps to reduce the mobility of macroradicals, limiting termination. Therefore, we can achieve a higher maximum reaction rate at higher initiator concentrations. On the other hand, with a further increase in its concentration, the opposite trend is observed. Above a certain concentration of a photoinitiator, the induction time decreases, the initial value of the reaction accelerates (maximum value of the second derivative of the rate with respect to time), the maximum reaction rate is reached at a lower conversion rate, and the total reaction time decreases [33,34].

Moreover, process conditions, such as temperature, atmosphere, and light intensity, also affect photopolymerization kinetics. An increase in irradiance and temperature leads to an increase in the reaction rate and the conversion of double bonds. High light intensity, around tens of mW/cm^2^ or more, leads to an increase in monomer mobility due to an increase in temperature.

Telechelic methacrylic macromonomers comprising dimer fatty acids derived from C18 unsaturated fatty acids, such as linoleic acid obtained from vegetable oils, have already been reported in the literature [35]. The properties of such dimer fatty acid derivatives can be tuned by ester, anhydride, or urethane bonds, and the obtained liquids are susceptible to photocuring reactions and the formation of flexible materials [36]. However, such macromonomers were synthesized with the use of organotin-based catalysts, which exhibit cytotoxicity and are difficult to remove from polymers [37]. Therefore, we recently developed ester–urethane macromonomers containing a long-chain fatty ester core by using bismuth and zinc organocatalysts. The obtained materials showed low toxicity after photocuring [38]. However, the photopolymerization kinetic of such networks has never been evaluated.

During photopolymerization, the properties of the system undergo much more rapid changes than during a thermally initiated reaction. The system, in the form of a liquid, transforms very quickly, often in less than one second, into a glassy, cross-linked polymer. The consequence of such rapid property changes is the unusual behavior of macromolecules. The reaction kinetics are controlled by diffusion, and the system exhibits forced nonequilibrium states with gradients in concentration, temperature, and radiant intensity. The resulting network is accountable for the autocatalytic effect, which hinders termination, consequently accelerating the polymerization process. Prout and Tompkins initially presented the kinetic equation for autocatalytic reactions in the solid state [39]. Later, Sestak and Berggen extended the formula, and Kamal was the first to apply it in polymer science to describe the crosslinking of polyester resins [40,41]. The Sestak–Berggen equation, designed for isothermal conditions (Equation (1)), facilitates the determination of the partial orders of reaction and the apparent reaction rate constant. This model is also applicable to nth-order systems. An autocatalyzed reaction follows the empirical relationship:

(1) $$\frac{d\alpha }{dt}=k\times \alpha^{m}{\left(1-\alpha \right)}^{n}$$

(2) $$k=Ze^{\frac{-E\alpha }{RT}}$$

where

*dα*/*dt*—reaction rate (1/s);
*k—*rate constant (1/s);
α—conversion degree;
*m*, *n*—rection orders;
*Eα—*activation Energy (J/mol);
*Z—*pre-exponential factor (1/s);
*T—*absolute temperature (K);
*R—*gas constant (8.314 J/mol × K).

This kinetic model is applied to evaluate the rate constant (*k*) and orders of the reaction (*m*, *n*). The Avrami equation is also employed to determine the *t*1/2, representing the time at which the system reached 50% of carbon–carbon double bond conversion. The general equation under isothermal conditions is given in Equation (3):

(3) $$\ln\left[-\ln\left(1-\alpha \right)\right]=\ln\left[k\right]+p\times ln[t]$$

where

*α—*conversion degree;
*k—*rate constant (1/s);
*p*—Avrami reaction order (often = 4);
*t—*time (s).

Hence, in this paper, we utilized differential scanning photo-calorimetry (photo-DSC) to examine the influence of parameters such as the concentration of photoinitiators, the type of atmosphere, and light intensity on the kinetic photopolymerization of ester–urethane polymer networks. By analyzing the aforementioned parameters, the photopolymerization process can be optimized to produce polymers with the desired features. The study also provides insights into the differences between different photoinitiators and how alterations in light intensity and atmosphere can affect the overall polymerization process. The composition containing telechelic methacrylate ester–urethane macromonomer, synthesized using zinc (II) acetyloacetonate (ZnAc) described in our earlier work [38], has been chosen for this study. Two different phosphine photoinitiators (known as type I photoinitiators) at different concentrations of 0.5, 1, and 2% by weight were investigated. The photo-DSC study was performed at a constant temperature of 37 °C (mimicking human body temperature conditions).

## 2. Materials and Methods

### 2.1. Materials

**Figure 1:**
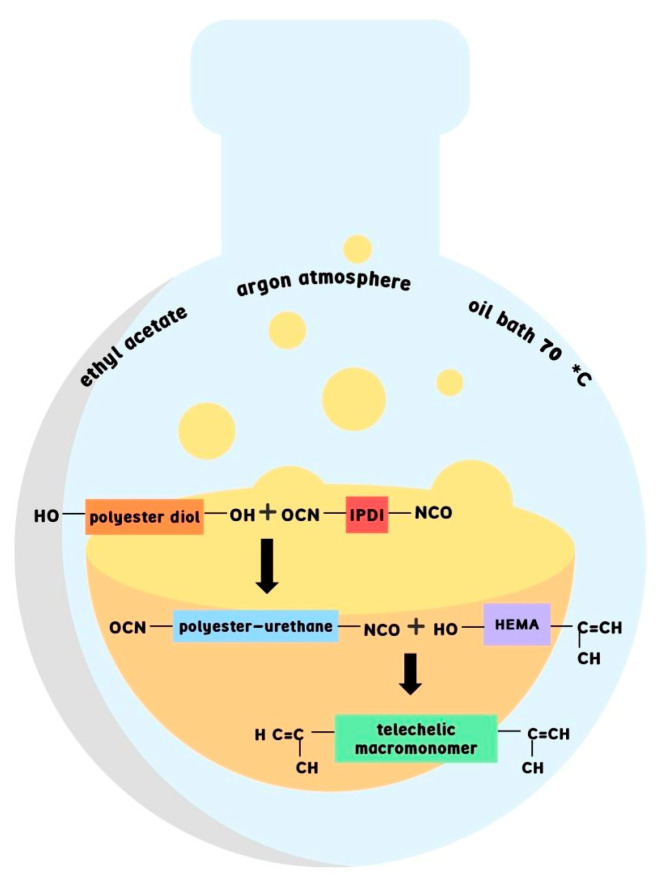
Scheme of the synthesis of telechelic macromonomer containing ester and urethane groups.

The telechelic methacrylate ester–urethane macromonomer was synthesized based on our previous work without modification [38]. Briefly, the two-step synthesis process involved using the polyester diol (Priplast 1838) provided by Cargill Bioindustrial (Gouda, The Netherlands), which is derived from vegetable oils. In the first step, polyester diol reacted with isophorone diisocyanate (98%) (IPDI, Merck KGaA (Darmstadt, Germany)) to create urethane groups. Next, 2-hydroxyethylmethacrylate (97%) (HEMA, Merck KGaA (Darmstadt, Germany)) was added to induce terminal methacrylic groups enabling photopolymerization (Figure 1). The synthesis was performed in the presence of zinc (II) acetyloacetonate (ZnAc, Sigma Aldrich, Poznań, Poland) catalyst, and before the second step, phenotiazine (PTZ, Sigma Aldrich, Poznań, Poland) was added. The synthesis was conducted in ethyl acetate (EtOAc) and purified by precipitation into methanol, both supplied by Chempur (Piekary Śląskie, Poland). The two-step reaction was carried out in an argon atmosphere at an elevated temperature (70 °C). The reaction progress was monitored using Fourier transform infrared spectroscopy (BRUKER ALPHA Platinum spectrometer (Bremen, Germany)) at room temperature (4000–600 cm^−1^, resolution 2 cm^−1^, 32 scans) by tracking the ratio between absorbance at 2262 cm^−1^, which corresponds to N=C=O vibration in decaying isocyanate groups of IPDI, and at 1526 cm^−1^, which corresponds to N-H vibrations of the formed urethane bonds. The FTIR spectroscopy was also used to characterize materials after photocuring. Two photoinitiators, Omnirad 819 (O819) and Omnirad 2022 (O2022), were supplied from IGM resins (Waldwick, The Netherlands). Reagents were used as received, except for HEMA, which was distilled under reduced pressure prior to use.

### 2.2. Characterization Methods

Proton and carbon nuclear magnetic resonance (^1^H and ^13^C NMR) spectra of the monomer in deuterated chloroform (CDCl_3_) were recorded using a Bruker DPX HD-400 MHz (Billerica, MA, USA) at 25 °C. The instrument was fitted with a 5 mm Z-gradient broadband decoupling inverse probe.

Molar masses (*M_z_*) of monomers were determined from iodine value according to ISO 3961 [42] and using Equation (4) according to our earlier work [35]

(4) $$M_{z}=\frac{50,760+f\times M_{1}{LI}_{1}}{{LI}_{2}}$$

where

*M_z_—*molar mass;
*f*—the average number of core moieties per one macromer molecule, as calculated from ^1^H-NMR;
*M*_1_—molar mass of the raw material (Priplast 1838);
*LI*_1_—iodine value of the raw material (Priplast 1838);
*LI*_2_—iodine value of the monomer.

Additionally, molecular weights and molecular weight distribution of the synthesized macromonomer were determined by gel permeation chromatography (GPC). The measurements were conducted at 35 °C using a Wyatt (Dernbach, Germany) instrument equipped with a guard column and four Perfect Separation Solutions (PSS) columns (50, 100, 1000, and 100,000 Å). WGE Dr. Bures Dn 2010 differential refractometer (RI) and Wyatt MALLS DAWN HELEOS multi-angle light scattering (LS) were employed as detectors. THF was used as an eluent with a flow rate of 1 mL/min.

Dynamic viscosity tests of macromonomers were performed using a BROOKFIELD AMETEK rotary rheometer (Middleboro, MA, USA). The measurement was carried out with the head in the plate–plate system with a diameter of ϕ = 40 mm, the distance between plates h = 1 mm, deformation of 30%, and a constant shear rate $\dot{𝛾}$ (1/s): 0.200. The measurements were performed at temperatures of 25 °C and 37 °C.

UV-DSC study was performed using the Q2500 DSC (TA Instruments, New Castle, DE, USA) equipped with optical accessory OmniCure 2000 Spot UV Curing System (with High-Pressure 200-Watt Mercury Vapor Short Arc). Samples of monomers weighing 5 mg were mixed with 0.5, 1, and 2% wt. of photoinitiators, Omnirad 819 and Omnirad 2022. Measurements were carried out at a wavelength of 365 nm in both atmospheres (inert argon and in air, at a flow rate of 20 mL/min) at 37 °C. Samples were irradiated at two different intensities, 20 and 50 mW/cm^2^, respectively. The maximum conversions (*C_max_*) were calculated based on the Equation (5):

(5) $$C_{max}=\frac{M\times ∆H}{n\times ∆H_{p}}$$

where

*M—*molar mass of the monomer;
Δ*H—*enthalpy (J/g);
*n—*the number of methacrylate groups in the molecule—2;
Δ*Hp*—the theoretical molar heat of polymerization (J/mole of methacrylate groups). The value of Δ*Hp* for methacrylate groups was assumed to be 58.6 kJ/mol [43]. Experimentally determined values of the heat of polymerization of methacrylate monomers are in the range of 45 to 60 kJ/mol [44,45,46].

Results were analyzed with TRIOS program, applying two isothermal models dedicated to autocatalytic systems, i.e., Sestak–Berggen and Avrami. All experiments have been repeated at least two times to ensure data reproducibility.

### 2.3. Photopolymerization of Elastomeric Films

The photopolymerization of ester–urethane macromonomers end-capped with methacrylic functionalities was carried out using a light-curing system in air and argon atmospheres, respectively. The samples were crosslinked for 3 min under two different light intensities: 20 mW/cm^2^ and 50 mW/cm^2^, respectively. Silicone molds with dimensions of 1 cm × 3 cm were used for sample preparation and then for the photopolymerization. The light-curing system with the specified light intensity was positioned at a fixed distance from the samples. The photopolymerization was initiated by exposing the samples to the LED light source of 385 nm wavelength.

**Figure 2:**
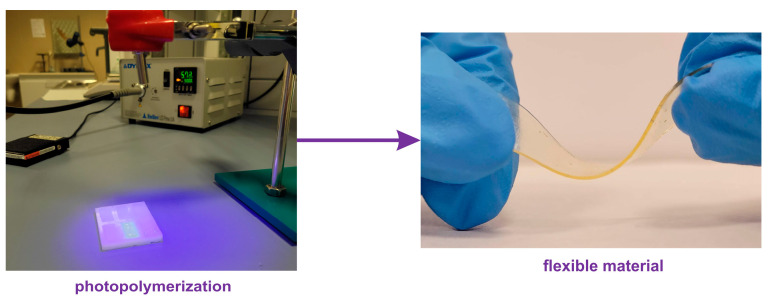
Photocuring of telechelic macromonomers with UV light towards flexible material (polymer network).

Additionally, photocured elastomeric thin films (0.5 mm) (Figure 2) irradiated by DYMAX Bluewave LED Prime UVA (Dynamax; Torrington, CT, USA) light source were subjected to Dynamic Mechanical Thermal Analysis (DMTA) with a Q800 DMTA instrument (TA Instruments; New Castle, DE, USA). The measurements were conducted at a frequency of 1 Hz and with an amplitude of 10 µm. The process began by cooling the samples to −90 °C, followed by subsequent heating to 120 °C with a heating rate of 3 °C per minute.

## 3. Results

### 3.1. Macromonomer Characterization

The chemical structure and degree of acrylation of macromonomers were confirmed by nuclear magnetic resonance (^1^H NMR) (Figure S1 in Supplementary Material), while the resulting polymer network was characterized with FT-IR spectroscopy (Figure S2 in Supplementary Material). Additionally, the *f* parameter, essential for the evaluation of molar mass from the iodine value measurements, was calculated from Equation (4) and equaled 1.06.

The molecular mass of the macromonomer was evaluated by gel permeation chromatography (GPC) and the iodine value (IV) measurement, and the viscosity of the monomer was measured at 25 °C and 37 °C. The results are shown in Table 1. The M_w_ was found to be 13,300 g/mol and the M_n_ was 7800 g/mol. Meanwhile, based on the iodine value, the calculated molar mass was 5426 g/mol. The results obtained from GPC always have considerably higher values. In our earlier work [35], we discussed the origin of these differences, as the linear structure of the used PS column is substantially different from the branched structures of the telechelic macromers, contributing to inaccurate results. Therefore, the molar masses obtained by IV measurements are more accurate and closer to the theoretical.

### 3.2. UV-DSC Study

The effect of Omnirad 2022 and Omnirad 819 photoinitiators concentration, the type of atmosphere, and UV light intensity on the macromonomers’ conversion and the reaction rate were investigated using photo-DSC. The results were analyzed for two different irradiation intensities, i.e., 20 mW/cm^2^ and 50 mW/cm^2^.

**Figure 3:**
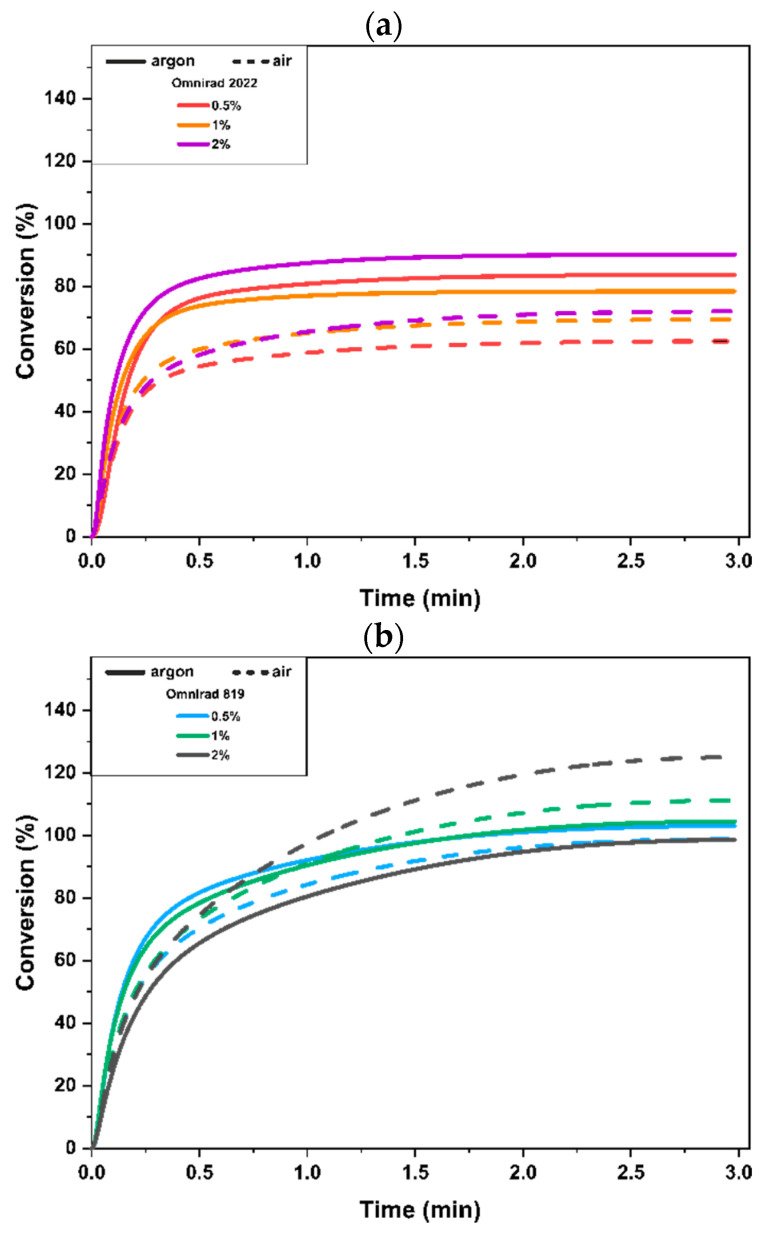
Monomer conversion as a function of time for different concentrations, 0.5, 1, and 2% wt., of photoinitiator Omnirad 2022 (**a**) and Omnirad 819 (**b**) at 20 mW/cm^2^.

Figure 3 shows changes in conversion in the function of time for macromonomers containing variable amounts of Omnirad 2022 (Figure 3a) and Omnirad 819 (Figure 3b) cured at the irradiation intensity of 20 mW/cm^2^. Overall, some of the calculated maximum conversions are above 100%, which is a result of the assumed approximate value of the heat of polymerization. Experimentally determined values of the heat of polymerization of methacrylate monomers can range from 45 to 60 kJ/mol, depending on the structure of the monomer [45,46,47]. According to the literature, conversion values higher than 100% were reported by Andrzejewska (for compositions with 2,2′-thiobisethanol diacrylate (TEDA) and 2,2′-oxybisethanol diacrylate (OEDA)) [48].

Photopolymerization in the presence of Omnirad 819 (Figure 3b) allowed for higher conversions in the air atmosphere compared to compositions containing Omnirad 2022 (Figure 3a). Interestingly, for the systems containing Omnirad 2022, a clear difference in conversion between the inert (argon) atmosphere and in the oxygenating air atmosphere can be observed. The significantly lower conversion in the air can be explained as a result of oxygen inhibition because oxygen causes the quenching of the excited states of the initiators, thereby reducing the efficiency of initiation.

**Figure 4:**
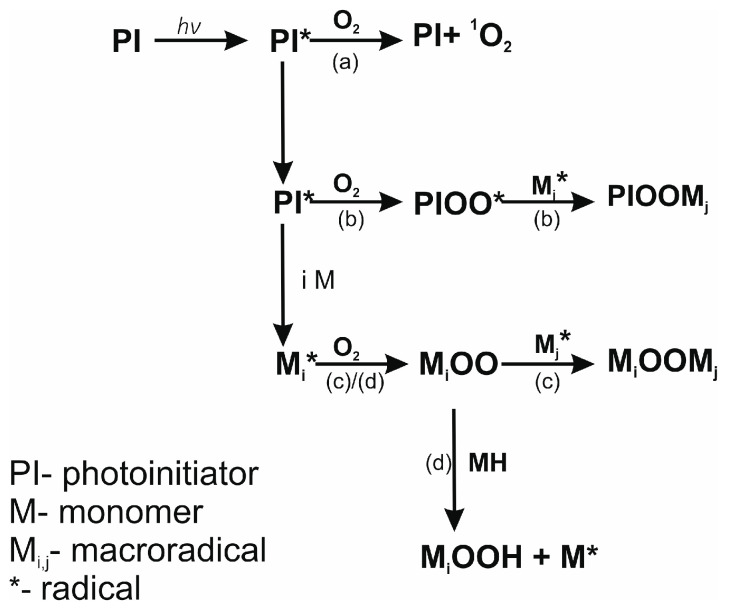
The main reactions in the photopolymerization process resulting from oxygen inhibition: (a) quenching of the excited state of the initiator, (b) and (c) premature termination of the kinetic chain, (d) hydrogen atom detachment by the superoxide radical and further propagation.

Moreover, oxygen reacts with promoting radicals and primary radicals to form superoxide radicals. Superoxide radicals are not highly reactive towards the monomer, while they easily react with other primary radicals and macroradicals. This leads to a decrease in the rate of propagation and an increase in the rate of termination, contributing to an unfavorable shortening of the kinetic chain. If hydrogen atom donors are present in the system, superoxide radicals can attach to a hydrogen atom and form a new radical capable of propagation (case (d) in Figure 4) [49].

The opposite effect of the oxygen presence on C_max_ is observed for the composition with Omnirad 819, where samples cured in air exhibited higher (or similar, at 0.5% of photoinitiator concentration) conversion in comparison to samples cured in argon.

Materials cured with Omnirad 2022 showed higher C_max_ with increasing concentrations of the photoinitiator, i.e., 1 and 2 mol%, and these changes were independent of the curing atmosphere. In the case of Omnirad 819 (Figure 3b), a similar trend is observed only for materials cured in an air atmosphere (the higher the concentration of the photoinitiator, the higher the conversion).

**Figure 5:**
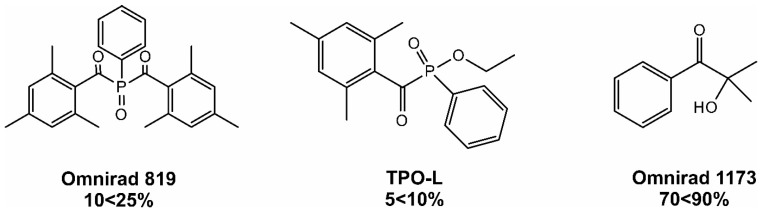
Chemical structure of Omnirad 819 and components of Omnirad 2022, which is a blend of Omnirad 819, TPO-L, and Omnirad 1173.

The observed differences can be explained by the chemical structure of photoinitiators. Both photoinitiators are type I photoinitiators. This means that the radicals are formed by homolytic decay of the excited state of the initiator excited by the light. Omnirad 819 is a trade name for bis(2,4,6-trimethylbenzoyl)-phenylphosphine oxide, while Omnirad 2022 is a blend that includes Omnirad 819 (up to 25%) and two other photoinitiators: TPO-L [ethyl phenyl(2,4,6-trimethylbenzoyl)phosphinate] and Omnirad 1173 (2-hydroxy-2-methyl-1-phenylpropanone). Their chemical structures are shown in Figure 5.

**Figure 6:**
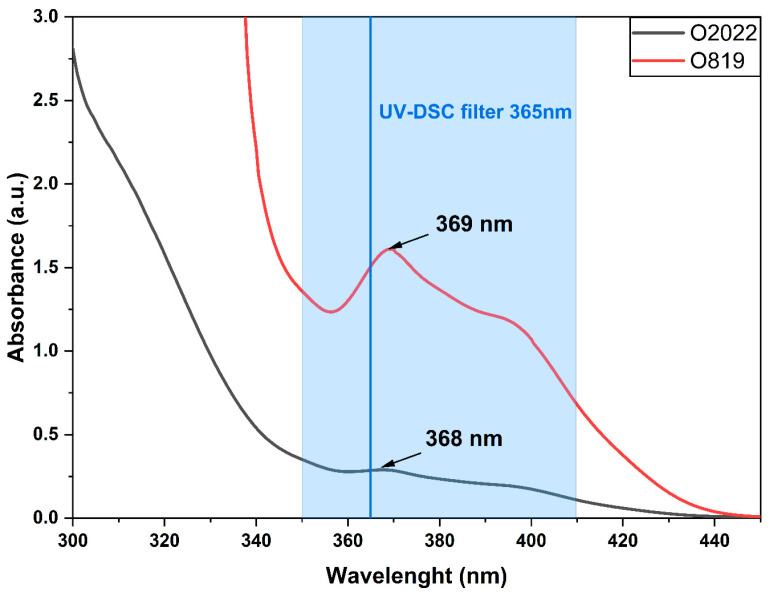
Spectra of photoinitiators performed for solutions of 0.1% in acetonitrile (according to the datasheet).

The used photoinitiators absorb the light in the wavelength range of 360–420 nm. The maximum adsorption of Omnirad 819 in acetonitrile, as reported by the manufacturer, is found at 380 nm, whereas our measurements indicated a maximum absorbance at 369 nm (Figure 6). The Omnirad 2022’s adsorption maximum, according to the manufacturer, is 370 nm, whereas the experimental results indicated the maximum to be 368 nm (Figure 6).

**Figure 7:**
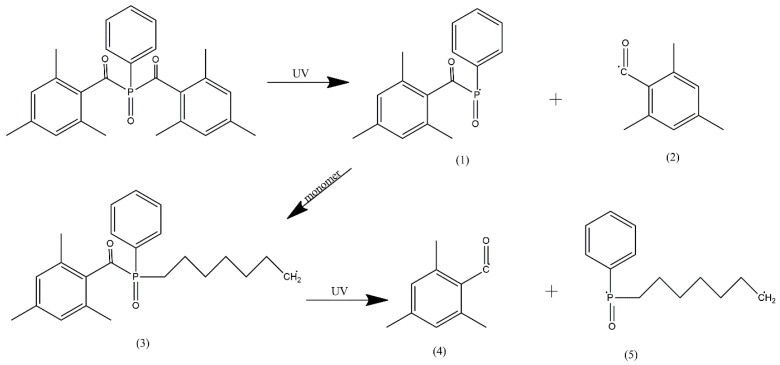
Two-step photolysis of Omnirad 819 [37].

Both Omnirad 819 and TPO-L (the component of Omnirad 2022) are phosphine oxides. Their photolysis (illustrated for Omnirad 819 in Figure 7) results in the formation of two radicals—phosphinoyl (1) and benzoyl (2) [50]. Both radicals initiate polymerization, but phosphinoyl radicals are more reactive towards vinyl monomers than any benzoyl radicals—the initiation rate constant is 1–2 orders of magnitude higher for them. After the phosphinoyl radical attaches to the monomer/oligomer (3), another breakdown can take place, resulting in the formation of a second benzoyl radical (4) and a larger diradical (5).

Despite the fact that compositions obtained with the use of this photoinitiator do not demonstrate surface tackiness, it, unfortunately, has a limited depth of cure compared to, for example, TPO-L [51]. To achieve a balance between a high degree of crosslinking on the surface and in deeper layers of the material, it is usually recommended to use mixtures of Omnirad 819 with other photoinitiators. Thus, Omnirad 2022 is such an example, where an acetophenone derivative, namely Omnirad 1173, is present. An advantage of incorporating another phosphine (TPO-L) photoinitiator is that one of the resulting radicals has a linear structure, and this much spatially smaller radical devoid of an aromatic ring will overcome steric hindrances more easily and reach the double bonds of the macromonomer with greater efficiency [32].

When comparing the differences in chemical structure of Omnirad 819 and Omnirad 2022, it is clear that in case of the latter, the mixture of Omnirad 1173 (70 < 90%) and TPO-L (5 < 10%) with Omnirad 819 (which constitute only 10 < 25%), has a direct translation to higher conversions at higher content of Omnirad 2022 in an inert atmosphere (Figure 3a).

Moreover, increasing the initiator content in argon results in the highest maximum reaction rate, R_max_, as can be seen in Figure S2 in Supplementary Material).

The higher concentration of the Omnirad 2022 photoinitiator (Figure S2) triggers a higher rate of initiation and the generation of more radicals at the beginning of the reaction, which accelerates propagation. The use of Omnirad 819 (Figure S2b) did not demonstrate such a correlation.

The shape of all R(C) curves (Figure S3 in Supplementary Material) indicates that auto-acceleration occurs from the beginning of the reaction. It is noticeable that the maximum reaction rate (R_max_) is achieved rapidly in the initial stage. Compositions with a blend (Omnirad 2022) accomplish R_max_ for lower conversions (peaks are narrower). Moreover, the values of R_max_ differ significantly for systems with different photoinitiators. The R_max_ values increase with the decrease in the photoinitiator for compositions with Omnirad 819. Moreover, the values are higher for an argon atmosphere, and the difference between the atmospheres is higher for lower concentrations of photoinitiator, whereas, for the highest concentration (2 mol% of Omnirad 819), R_max_ values are equal regardless of atmosphere. On the other hand, the R_max_ values increase with the increase in Omnirad 2022; the influence of the atmosphere is major and independent of photoinitiator concentration.

The maximum reaction rate for compositions containing Omnirad 2022 is much higher than for systems with Omnirad 819. The difference may be attributed to the structure and spectral range of the photoinitiators. The study used a 365 nm filter, and the absorption maximum of Omnirad 2022 is nearer to this value (according to the manufacturer: 370 nm; measured on our apparatus: 368 nm), while the absorption maximum of Omnirad 819 is more distant (and from the information sheet, it appears to fall at 380 nm; measured on our apparatus: 369 nm) (Figure 6). It also leads to the conclusion that a higher reaction rate results in a lower maximum conversion. This might be explained by analyzing the values of the reaction order, *n* (Table 1). High values of *n* (above 2) indicate termination occurring due to high viscosity (limited free diffusion in the system). In other words, for systems that have reached a higher maximum reaction rate, higher values of *n* are observed. The rapid increase in the viscosity of the system, and thus the limitation of free diffusion described by the *n*th order of the reaction, caused the reaction to slow down, thus triggering lower maximum conversions. Additionally, a comparison of conversion at the maximum reaction rate (Cu) shows slightly higher values for systems with Omnirad 2022 than for Omnirad 819.

Considering Figure 3a, Figures S2a and S3a (systems with Omnirad 2022), it can be noticed that a higher concentration of photoinitiator leads to higher reaction rates. This might be assumed as a result of the higher number of formed radicals. On the other hand, compositions containing Omnirad 819, as shown in Figure 3b, Figures S2b and S3b, exhibit only slight differences between the 0.5% and 1% concentrations. Furthermore, unusual phenomena were observed for a higher concentration of photoinitiator Omnirad 819, which leads to significantly lower R_max_.

**Figure 8:**
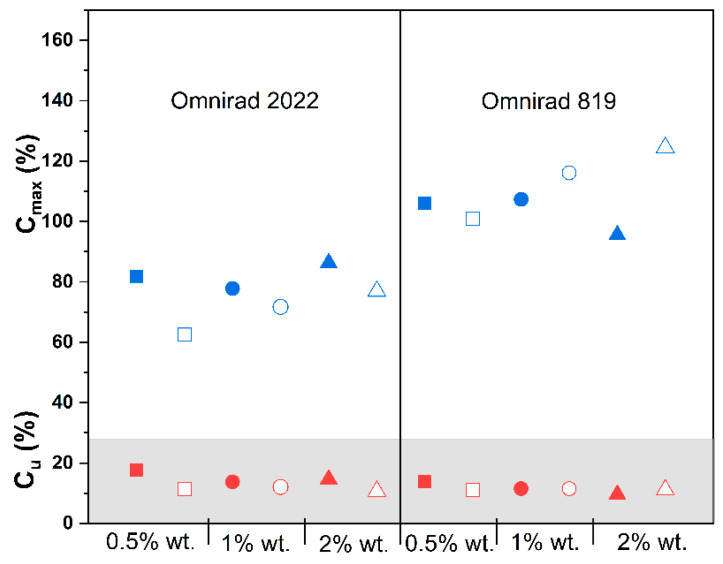
Influence of photopolymerization conditions on the conversion value at the highest reaction rate (Cu, in gray, red symbols) and maximum conversion (*C_max_*, blue symbols) for reactions in argon (closed symbols) and air atmospheres (open symbols) at 20 mW/cm^2^. Square—0.5% wt.; bullet—1% wt.; triangle—2% wt.

**Figure 9:**
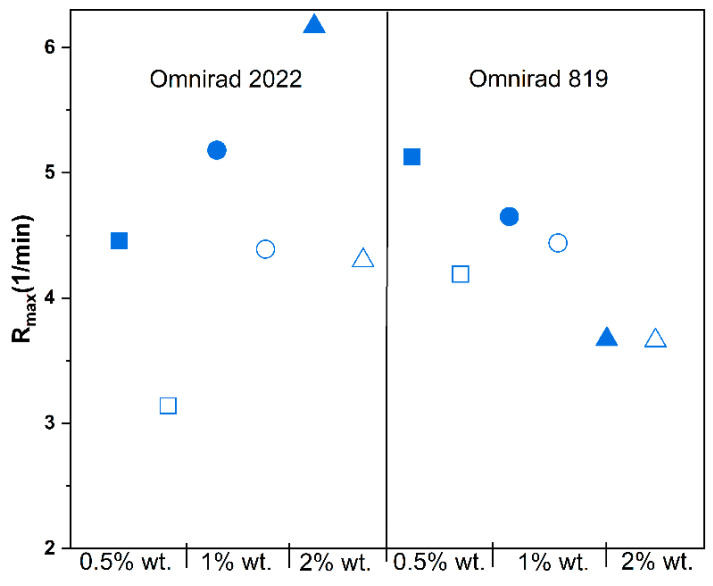
Effect of photopolymerization conditions on the maximum reaction rate (*R_max_*) for reactions in argon (closed symbols) and air atmospheres (open symbols) at 20 mW/cm^2^. Square—0.5% wt.; bullet—1% wt.; triangle—2% wt.

**Figure 10:**
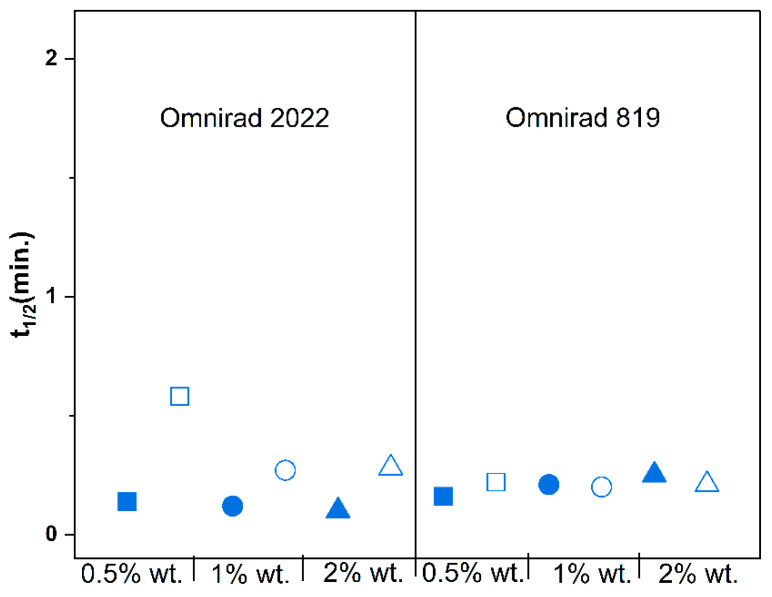
Effect of photopolymerization conditions on the time to reach 50% conversion (t_1/2_) for reactions in argon (closed symbols) and air atmospheres (open symbols) at 20 mW/cm^2^. Square—0.5% wt.; bullet—1% wt.; triangle—2% wt.

Moreover, in Figure 8, Figure 9 and Figure 10, significant differences between the types of curing atmosphere are noticeable. The highest maximum reaction rates of 6.17 min^−1^ were achieved for the sample containing 2% wt. of Omnirad 2022 cured in argon, in comparison with a value of 4.36 min^−1^ for the same sample cured in air. The major influence of oxygen inhibition is observed for systems with Omnirad 2022; the difference is smaller for systems with Omnirad 819, especially with the increased concentration of photoinitiator (Figure S3 in Supplementary Material). Therefore, it might be concluded that both or one of the other chemical compounds (TPO-L or/and Omnirad 1173) from the Omnirad 2022 blend (see structure in Figure 5) are/is more sensitive to oxygen molecules.

Higher maximum conversions were obtained for compositions with Omnirad 819. The differences between the same compositions cured in air and argon are minor, except for the highest photoinitiator concentration, where a noticeably higher C_max_ was obtained for samples cured in an atmosphere rich in oxygen. Contrary to this, maximum conversions of compositions with Omnirad 2022 depend on the presence of oxygen in the curing atmosphere (higher C_max_ were obtained for compositions cured in argon).

Oxygen inhibition influences the t1/2 (Figure S3 in Supplementary Material) but mainly influences systems cured with Omnirad 2022. Moreover, the increase in the photoinitiator concentration minimizes the effect of oxygen inhibition, which is related to the generation of more radicals. In summary, the systems cured with Omnirad 2022 are more susceptible to oxygen inhibition when cured with a light intensity of 20 mW/cm^2^ than those cured with Omnirad 819.

The kinetic parameters of photocrosslinking are presented in Table 2. The rate constant, k, is related to the reactivity of the system, the type and concentration of the initiator, the reaction atmosphere, light intensity, and temperature. At a constant temperature and in the same type of reaction atmosphere, a comparison of the *k* values provides information about the relative reactivity of the photoinitiator. Significantly higher values (from 0.68 to 0.38) were obtained for Omnirad 2022 compared to Omnirad 819 (from 0.26 to 0.11). Surprisingly, an increase in photoinitiator concentration leads to a lower *k*. This can be explained by the higher reactivity of the blend (Omnirad 2022) than the Omnirad 819 single component. The effect of the atmosphere on the reaction rate constant is negligible.

The reaction order *m* values are also summarized in Table 2. A higher value of *m* indicates a stronger effect of autoacceleration on the kinetics of the photopolymerization, and zero if the reaction does not fit into the autoacceleration model. Autoacceleration is induced by a decrease in the rate of the termination reaction. The value of the reaction order, *m*, for all compositions is above 0, supporting the autoacceleration mechanism of the photopolymerization and justifying the choice of the kinetic models. Furthermore, the same trend is observed for reaction order *m* as for the reaction rate constant, *k*. The highest values are observed for Omnirad 2022, and the lowest are observed for concentrations of the photoinitiator.

The reaction order values, *n*, and the information they provide were discussed in detail to explain the differences in *C_max_*. Generally, for all of the systems, values are above 2 (except for value 1.73 found for 2 wt.% of Omnirad 819 in air). As for other kinetic parameters calculated based on the Sestak–Berggren model, oxygen inhibition is mainly observed for Omnirad 2022 and lower concentrations of photoinitiators (lower number of generated radicals).

In our work, we also investigate the effect of higher light intensity (50 mW/cm^2^) on the maximum conversion of the systems cured in an air atmosphere and in an inert gas.

**Figure 11:**
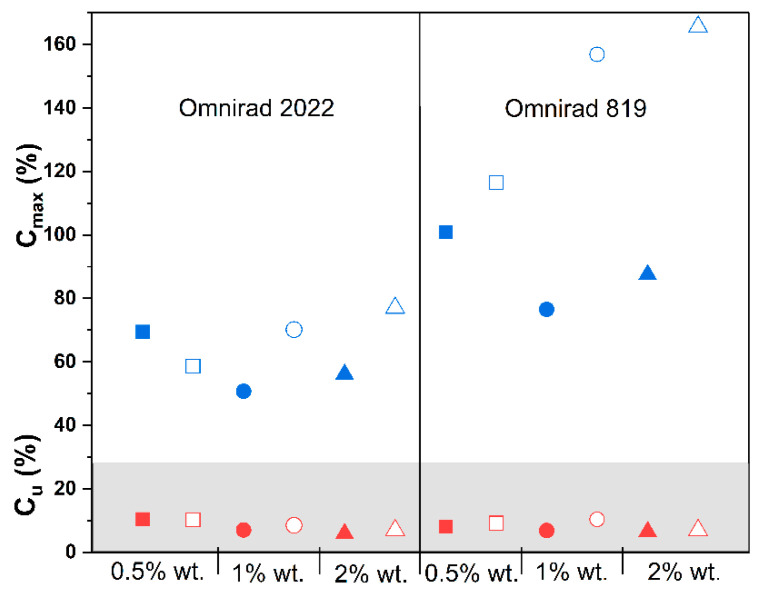
Influence of photopolymerization conditions on the conversion value at the highest reaction rate (Cu, in gray, red symbols) and maximum conversion (*C_max_*, blue symbols) for reactions in argon (closed symbols) and air atmospheres (open symbols) at 50 mW/cm^2^. Square—0.5% wt.; bullet—1% wt.; triangle—2% wt.

**Figure 12:**
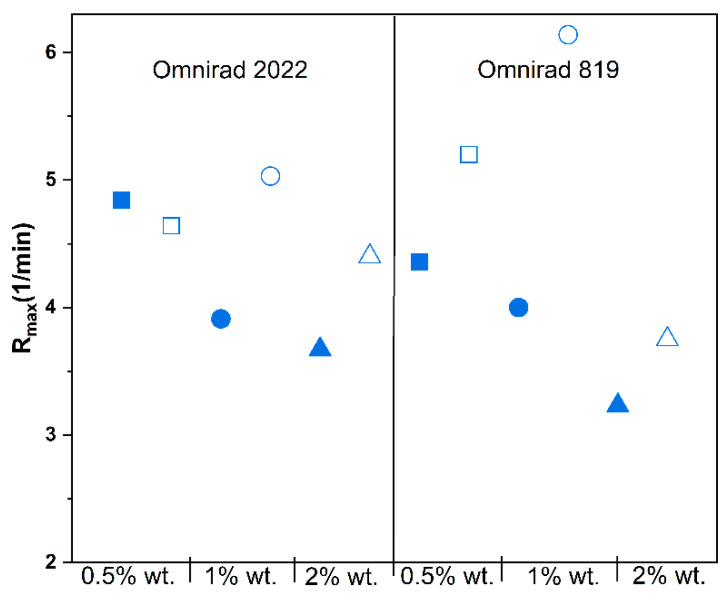
Effect of photopolymerization conditions on the maximum reaction rate (*R_max_*) for reactions in argon (closed symbols) and air atmospheres (open symbols) at 50 mW/cm^2^. Square—0.5% wt.; bullet—1% wt.; triangle—2% wt.

**Figure 13:**
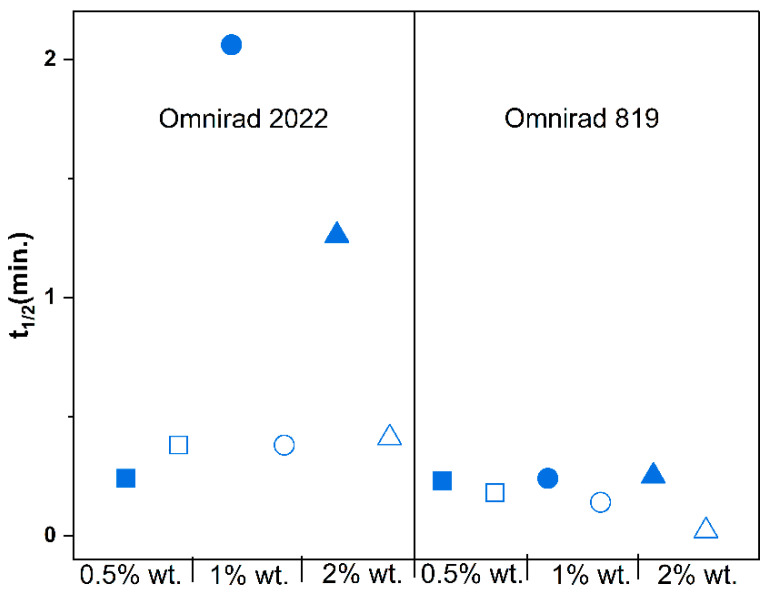
Effect of photopolymerization conditions on the time to reach 50% conversion (t_1/2_) for reactions in argon (closed symbols) and air atmospheres (open symbols) at 50 mW/cm^2^. Square—0.5% wt.; bullet—1% wt.; triangle—2% wt.

**Figure 14:**
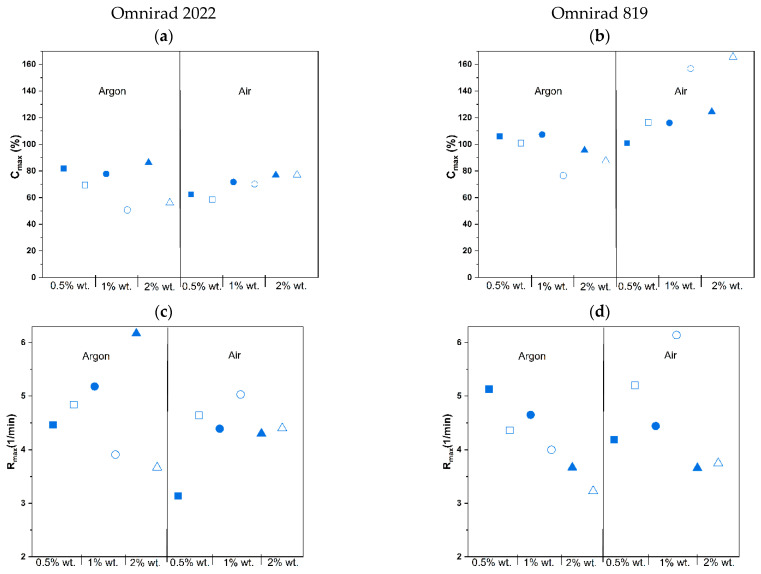
Effect of intensity of light on the maximum conversion (*C_max_*) (**a**) for Omnirad 2022 and (**b**) for Omnirad 819; maximum reaction rate (*R_max_*) (**c**) for Omnirad 2022 and (**d**) for Omnirad819. The reactions at 20 mW/cm^2^ (closed symbols) and 50 mW/cm^2^ (open symbols). Square—0.5% wt.; bullet—1% wt.; triangle—2% wt.

A higher concentration of photoinitiator Omnirad 819 led to higher C_max_ (Figure 11, Figure S4 in Supplementary Material) and lower R_max_ (Figure 12, Figure S5 in Supplementary Material), regardless of the curing atmosphere. On the contrary, compositions with Omnirad 2022 exhibited diverse patterns when exposed to various photoinitiator concentrations and atmospheric conditions. In an argon atmosphere, higher concentrations of Omnirad 2022 led to lower C_max_ (for 0.5% wt., around 70%; 1 and 2% wt. similar over 50%) and lower R_max_ (Figure S6 in Supplementary Material). On the other hand, in the air atmosphere, an increase in the photoinitiator concentration led to higher C_max_ (0.5% wt., around 69%; 1% wt. 70% and 2% wt. 77%). It might be a consequence of oxygen inhibition (formed radicals reacting with oxygen, creating peroxyl radicals that are less active towards the monomer). Thanks to that, the reaction is slower, and the number of free radicals is smaller in comparison to the same compositions cured in argon. It leads to a decrease in viscosity and a higher diffusion, thus resulting in the prolonged propagation of polymerization. The reaction order, *n* (Figure 13 and Figure 14, Table 3), of compositions cured with Omnirad 2022 supports this hypothesis. A similar situation is observed for epoxy systems, where the reaction rate is intentionally slowed down in order to obtain higher conversions [52]. In contrast to the results obtained for a light intensity of 20 mW/cm^2^ (Figure S3 in Supplementary Material), the R_max_ decreases with an increase in Omnirad 2022 content.

**Figure 15:**
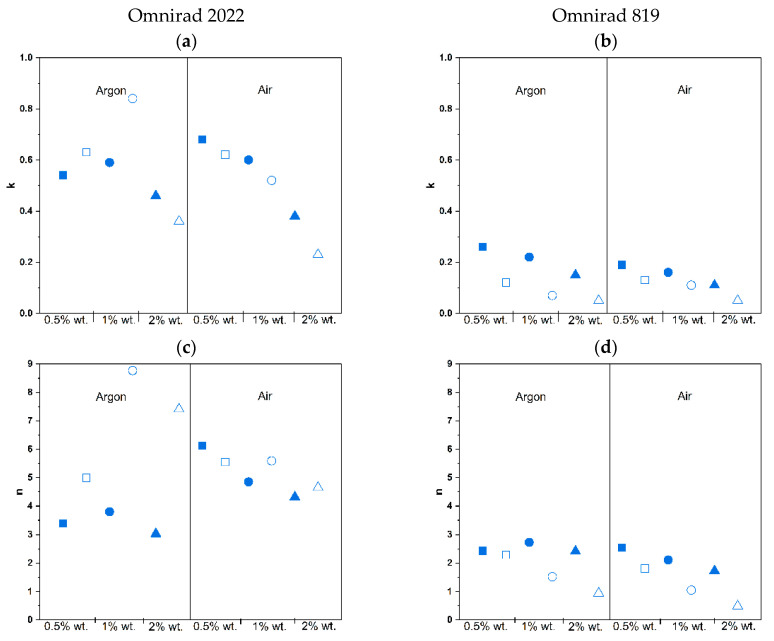

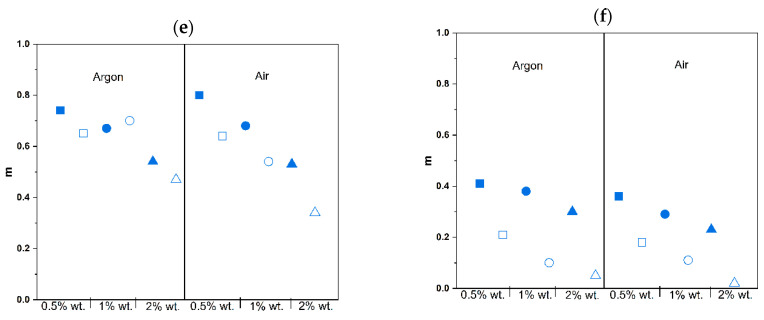
Effect of intensity of light on the rate constant *k* ((**a**) for Omnirad 2022 (**b**) for Omnirad 819), and reaction orders *n* ((**c**) for Omnirad 2022 (**d**) for Omnirad 819), *m* ((**e**) for Omnirad 2022 (**f**) for Omnirad 819) for reactions at 20 mW/cm^2^ (closed symbols) and 50 mW/cm^2^ (open symbols). Square—0.5% wt.; bullet—1% wt.; triangle—2% wt.

The kinetic parameters of the photocrosslinking process conducted with the use of light intensity 50 mW/cm^2^ are presented in Table 3 and Figure 15. The same pattern is observed for a lower light intensity. Higher values of *k* were obtained for Omnirad 2022 compared to Omnirad 819, and an increase in photoinitiator concentration leads to lower *k*. The obtained value of the reaction order, *m*, for all the systems is also above 0, but all values are lower than those obtained for the compositions cured at 20 mW/cm^2^ light intensity.

Moreover, the reaction order, *n*, values are also high. Compositions with the Omnirad 2022 are also above 2 and higher than those cured with lower light intensity. Additionally, the presence of an oxygen atmosphere decreases the values for both systems. As for Omnirad 819, the *n* values are lower (below 2) and even lower than those obtained for lower light intensity. This can explain the reason for larger conversions for compositions with Omnirad 819 that were attained with increased light intensity.

The light intensity influenced most of the presented parameters [53]. The maximum conversion of systems cured in an air atmosphere increases with higher light intensity (Figure 14, Tables S1 and S3 in Supplementary Material). Surprisingly, the C_max_ of compositions with Omnirad 2022 cured in argon decreases with the increase in light intensity, similar to R_max_, whereas *n* and *m* reaction orders are increasing. Therefore, it may be concluded that not only photoinitiator concentration but also light intensity influences the number of created radicals. Due to the presence of oxygen and the inhibition mechanism (Figure 4), systems cured in air led to higher C_max_ and R_max_ but lower *n* and m reaction orders. Oxygen reacted with free radicals, thus reducing the viscosity of the systems and extending photopolymerization times with lower maximum reaction rates.

Photocrosslinking of acrylic systems is always accompanied by the release of energy in the form of heat (exothermic reaction), which causes an increase in temperature [54,55,56]. Usually, during these reactions enforced nonequilibrium states, gradients in concentration, temperature, and radiation intensity occur. The temperature can increase significantly even up to 30–60 °C (from room temperature) [40].

**Figure 16:**
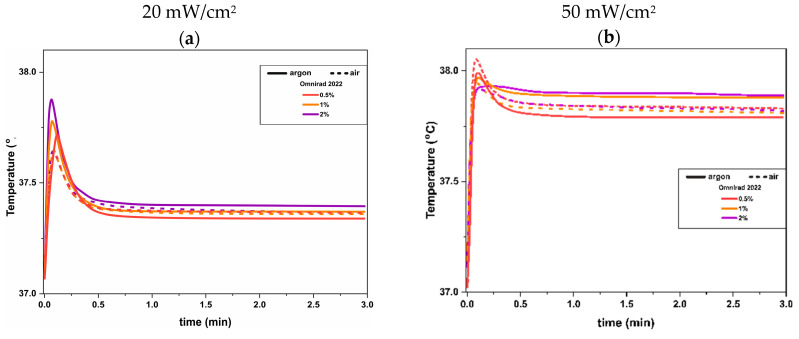

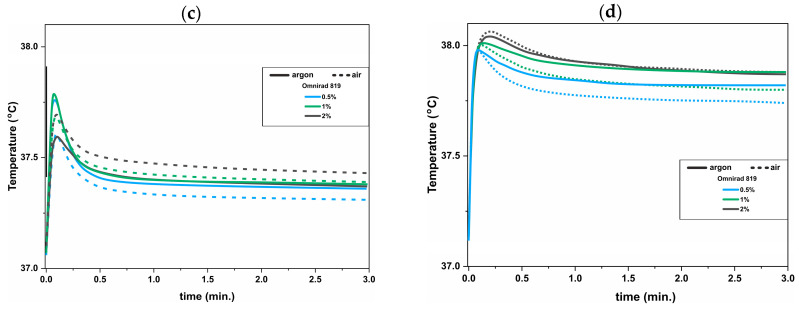
Temperature profiles of photocurable systems as a function of time (**a**,**c**) at 20 mW/cm^2^ and (**b**,**d**) at 50 mW/cm^2^.

The maximum temperatures of investigated systems are presented in Figure 16 and Tables S2 and S4 in Supplementary Material. It can be noticed from Figure 16 that when samples were cured at 20 mW/cm^2^, the temperature increased slightly (maximum from 37 °C to 38.1 °C). A much higher difference is visible for systems cured at higher light intensity. First of all, the shape of the plots is different. When samples are cured at 20 mW/cm^2^, the visible peak can be observed in the early stage (like the time-dependent reaction rate plot). After around 0.5 min, the temperature decreases to ~37.4 °C. On the contrary, for systems cured with higher light intensity, the peaks appear faintly. The maximum temperatures are slightly higher (~38.1 °C) and remain almost constant at ~37.9 °C). Thus, it can be concluded that an irradiance of 20 mW/cm^2^ is safe and does not exceed 37.5 °C independently from photoinitiator concentration, which is important if such materials will be suggested for medical applications.

### 3.3. DMTA

**Figure 17:**
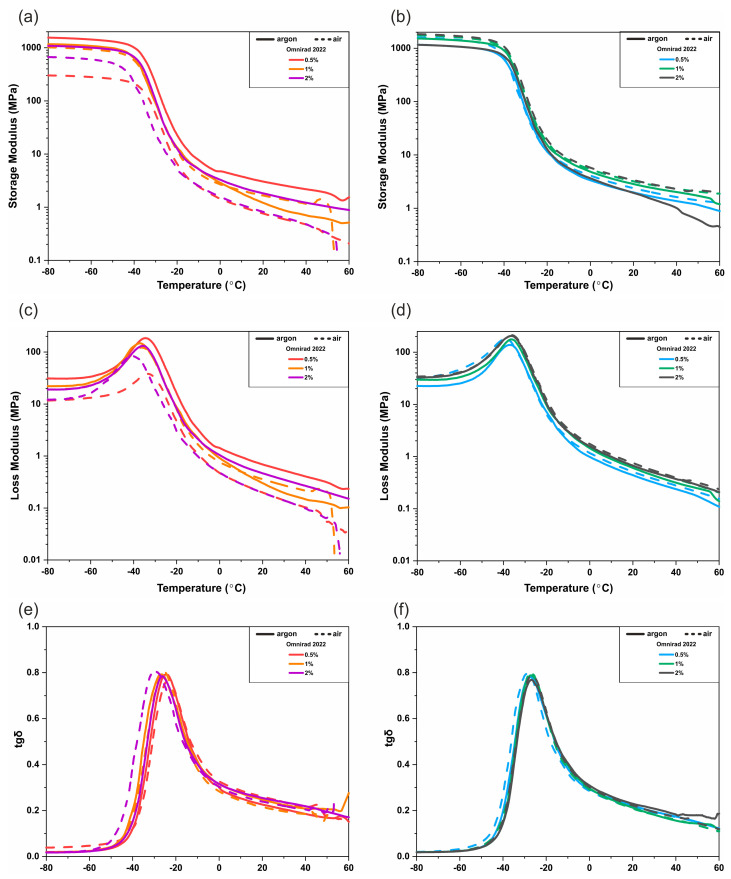
DMTA analysis of photocured polymer networks with use of 20 mW/cm^2^ light intensity: (**a**,**b**) the storage modulus (E′), (**c**,**d**) loss modulus (E″), and (**e**,**f**) the tangent of the phase angle (tan δ).

**Figure 18:**
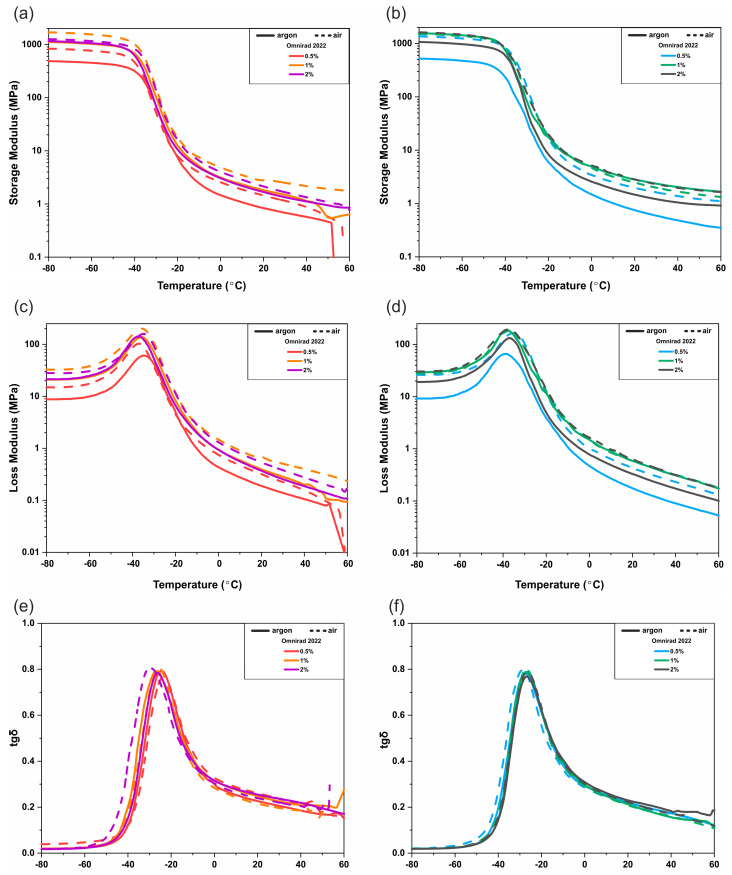
DMTA analysis of photocured polymer networks with the use of 50 mW/cm^2^ light intensity: (**a**,**b**) the storage modulus (E′), (**c**,**d**) loss modulus (E″), and (**e**,**f**) the tangent of the phase angle (tan δ).

To evaluate the effect of photocuring parameters on the resulting films’ properties, dynamic thermomechanical analysis (DMTA) has been performed. The results are presented in the form of temperature-dependent changes of storage modulus (E′), loss modulus (E″), and the tangent of the phase angle (tan δ) (Figure 17 and Figure 18 and Table S5). As can be noticed for materials cured with a light intensity of 20 mW/cm^2^, the glass transition temperature, T_g_, change is different for various photoinitiators (Figure 17). The T_g_ increases with the increase in Omnirad 819 concentration and with the decreased concentration of Omnirad 2022. As was already discussed (Figure 14a,b) in the previous chapter for Omnirad 2022, samples cured in argon reach higher C_max_ than those cured in air, which is opposite to samples cured with the use of Omnirad 819. The same trend can be observed for T_g_ values (Figure 17 and Figures S7 and S8 in Supplementary Material). In the case of samples cured with the use of higher light intensity, the influence of the atmosphere is more visible. The glass transition temperature of samples cured with the use of Omnirad 2022 increases with the decrease in photoinitiator concentrations only for samples cured in air, which is the opposite of the trend observed for an argon atmosphere (Figure 18). Those results also correspond to C_max_ and support the previously presented thesis that in systems with high concentrations of catalysts, higher light intensity, and within an inert atmosphere, a greater number of radicals is generated, which results in the premature termination of photopolymerization due to limited free diffusion.

Moreover, significant differences in values of storage modulus in the region below −20 °C are present (~300 to ~1400 MPa for Omnirad 2022 and ~600 to ~1700 MPa for Omnirad 819 at 20mW/cm^2^ and at 20 mW/cm^2^ ~500 to ~1700 MPa; ~500 to ~1600 MPa, respectively). Additionally, the comparison of E′, E″, and tan δ values at 37 °C has been deeply analyzed. The results of the storage modulus, loss modulus, and tangent of the phase angle are presented in Table S5 in Supplementary Material. The tan δ values are similar for all the samples and are in the range of 0.16–0.22 MPa, regardless of photoinitiator type, concentration, or light intensity. The curing parameters have a significant influence on the moduli values (Figure S9 in Supplementary Material). Generally, materials cured with Omnirad 819 show higher E′ and E″ when cured in air and with higher content of photoinitiator, regardless of light intensity. It might be the result of the previously described effect of oxygen inhibition, which causes a decrease in free radicals and leads to a decrease in viscosity, thanks to which premature termination did not occur. Interestingly, for the blend (Omnirad 2022), a similar trend is observed only for monomers cured with the use of higher light intensity (50 mW/cm^2^). For samples cured at 20 mW/cm^2^, the air atmosphere had the opposite effect. The oxygen presence results in lower E′ and E″ and lower C_max_. Furthermore, the sample with the lowest photoinitiator concentration (0.5% wt.) cured in argon is significantly more elastic (higher E′ and E″); nevertheless, that higher C_max_ was obtained for 2% wt. of Omnirad 2022. It might be connected with the presence of an optimal amount of uncured residuals in the crosslinked network, which most likely act as lubricants and simplify chain mobility.

The influence of lamp differences (photo-DSC mercury-vapor lamp, curing for DMTA with the use of LED lamp) is imperceptible. Both commercially available photoinitiators are suitable for curing with the use of both types of lamps. The negative oxygen inhibition effect appeared only for samples cured with Omnirad 2022 at lower light intensity (20 mW/cm^2^). The increase in light intensity neutralizes this negative effect.

## 4. Conclusions

We report here the kinetic studies of photocurable monomers using two type I photoinitiators. The influence of the photoinitiator chemical structure, its concentration, type of atmosphere, and light intensity were tested. Oxygen inhibition depends on photoinitiator type and light intensity, which are directly related to the number of radicals created in the first seconds of irradiation. An increase in the light intensity (from 20 to 50 mW/cm^2^) allowed us to obtain a higher maximum conversion in an air atmosphere regardless of the photoinitiator. Hence, the higher maximum conversion was reached at 50 mW/cm^2^. Generally, a higher photoinitiator concentration leads to higher maximum conversion, but a lower maximum reaction rate was explained by the limited diffusion of radicals in systems with high R_max_ due to the high viscosity.

Increasing the concentration of the photoinitiator accelerates the initiation rate and the generation of more radicals at the beginning of the reaction. However, beyond a certain concentration, 0.5% in this case, an opposite trend is observed, with a decrease in the induction time and the total reaction time.

The DMTA experiment revealed that the tan δ values of cured films are not affected by the photopolymerization parameters (light intensity, photoinitiator concentration, oxygen presence). On the other hand, the storage modulus and loss modulus values strongly depend on those parameters. The highest elasticity was not observed for samples with the highest maximum conversion but for film obtained by curing the composition containing 0.5% of Omnirad 2022 and in an argon atmosphere. This is likely due to the higher content of uncured residuals in samples that showed lower conversion.

In conclusion, the photoinitiator chemical structure and its concentration, light intensity, and the curing atmosphere showed a strong effect on the kinetic of methacrylate end-capped ester–urethane macromonomers. Higher light intensity, regardless of the photoinitiator type, leads to higher maximum conversion in the air atmosphere. Moreover, a negligible increase in temperature during the photopolymerization was noticed (~1 °C), which makes these systems very promising for medical applications.

## Figures and Tables

**Table 1:** Dynamic viscosity and GPC results for the synthesized macromonomer.

${\bar{M}}_{n}$ (from GPC) (g/mol)	${\bar{M}}_{w}$ (from GPC) (g/mol)	$Ð\;[{\bar{M}}_{w}/{\bar{M}}_{n}]$ (from GPC)	Molecular Mass (from IV) (g/mol)	Dynamic Viscosity at 37 °C (Pa×)	Dynamic Viscosityat 25 °C (Pa × s)
7800	13,300	1.69	5426	170 ± 4	571 ± 17

**Table 2:** Kinetic parameters of the Sestak–Berggren model for photocrosslinking at a light intensity of 20 mW/cm^2^.

Photoinitiator	Concentration of Photoinitiator (% wt.)	Atmosphere	k	n	m
Omnirad 2022	0.5	Argon	0.54 ± 0.07	3.39 ± 0.25	0.74 ± 0.03
		Air	0.68 ± 0.21	6.12 ± 0.24	0.80 ± 0.08
	1	Argon	0.59 ± 0.03	3.80 ± 0.07	0.67 ± 0.01
		Air	0.60 ± 0.01	4.85 ± 0.38	0.68 ± 0.02
	2	Argon	0.46 ± 0.02	3.03 ± 0.45	0.54 ± 0.06
		Air	0.38 ± 0.01	4.32 ± 0.68	0.53 ± 0.02
Omnirad 819	0.5	Argon	0.26 ± 0.00	2.43 ± 0.20	0.42 ± 0.00
		Air	0.19 ± 0.02	2.55 ± 0.06	0.36 ± 0.01
	1	Argon	0.22 ± 0.03	2.73 ± 0.36	0.38 ± 0.02
		Air	0.16 ± 0.01	2.11 ± 0.24	0.29 ± 0.02
	2	Argon	0.15 ± 0.05	2.42 ± 0.09	0.30 ± 0.05
		Air	0.11 ± 0.01	1.73 ± 0.02	0.23 ± 0.03

**Table 3:** Kinetic parameters of the Sestak–Berggren model for photocrosslinking at a light intensity of 50 mW/cm^2^.

Photoinitiator	Concentration of Photoinitiator (% wt.)	Atmosphere	k	n	m
Omnirad 2022	0.5	Argon	0.63 ± 0.03	4.99 ± 0.45	0.65 ± 0.02
		Air	0.62 ± 0.02	5.55 ± 0.86	0.64 ± 0.01
	1	Argon	0.84 ± 0.14	8.77 ± 1.88	0.70 ± 0.05
		Air	0.52 ± 0.01	5.59 ± 0.08	0.54 ± 0.00
	2	Argon	0.36 ± 0.04	7.41 ± 0.06	0.47 ± 0.04
		Air	0.23 ± 0.04	4.65 ± 0.72	0.34 ± 0.03
Omnirad 819	0.5	Argon	0.12 ± 0.02	2.29 ± 0.04	0.21 ± 0.03
		Air	0.13 ± 0.02	1.81 ± 0.34	0.18 ± 0.01
	1	Argon	0.07 ± 0.01	1.52 ± 0.23	0.10 ± 0.05
		Air	0.11 ± 0.04	1.05 ± 0.02	0.11 ± 0.03
	2	Argon	0.05 ± 0.01	0.94 ± 0.01	0.05 ± 0.03
		Air	0.05 ± 0.00	0.49 ± 0.04	0.02 ± 0.01

## Data Availability

Data are contained within the article and Supplementary Materials.

## Supplementary Materials

The following supporting information can be downloaded at: https://www.mdpi.com/article/10.3390/ma16237348/s1, Figure S1: ^1^H NMR of ester–urethane macromonomer; Figure S2: Reaction rate as a function of time for different concentrations 0.5, 1, and 2% wt. of photoinitiator Omnirad 2022 (a) and Omnirad 819 (b) at 20 mW/cm^2^; Figure S3: Reaction rate as a function of conversion for different concentrations 0.5, 1, and 2% wt. of photoinitiator Omnirad 2022 (a) and 819 (b) at 20 mW/cm^2^; Figure S4: Monomer conversion as a function of time for different concentrations 0.5, 1 and 2% wt. of photoinitiator Omnirad 2022 (a) and 819 (b) at 50 mW/cm^2^; Figure S5: Reaction rate as a function of time for different concentrations 0.5, 1, and 2% wt. of photoinitiator Omnirad 2022 (a) and 819 (b) at 50mW/cm^2^; Figure S6: Reaction rate as a function of conversion for different concentrations 0.5, 1, and 2% wt. of photoinitiator Omnirad 2022 (a) and 819 (b) at 50mW/cm^2^; Figure S7: Glass transition temperature of samples cured in argon (closed symbols) and air atmospheres (open symbols) with the use of 20 mW/cm^2^ light intensity; Figure S8: Glass transition temperature of samples cured in argon (closed symbols) and air atmospheres (open symbols) with the use of 50 mW/cm^2^ light intensity; Figure S9: Storage (blue) and loss (red) modulus at 37 °C of samples cured in argon (closed symbols) and air atmospheres (open symbols); Table S1: Parameters of the photopolymerization process at a light intensity of 20 mW/cm^2^; Table S2: Kinetic parameters of the Sestak–Berggren model for photocrosslinking at a light intensity of 20 mW/cm^2^; Table S3: Parameters of the photopolymerization process at a light intensity of 50 mW/cm^2^; Table S4: Kinetic parameters of the Sestak–Berggren model for photocrosslinking at a light intensity of 50 mW/cm^2^; Table S5: DMTA results.
