# Supplementary material for: Influence of Photoinitiator Type and Curing Conditions on the Photocuring of Soft Polymer Network
Source: Materials (Basel). 2023 Nov 25;16(23):7348. doi: 10.3390/ma16237348 (PMC10707501; doi:10.3390/ma16237348)
Supplement: Supplementary file 1 [file materials-16-07348-s001.zip › materials-2707932-supplementary.pdf]

Influence of photoinitiator type and curing conditions on the photocuring of soft polymer network

Malwina Niedźwiedź, Gokhan Demirci, Nina Kantor-Malujdy, Miroslawa El Fray*

Department of Polymer and Biomaterials Science, Faculty of Chemical Technology and Engineering, West Pomeranian University of Technology in Szczecin, Al. Piastow 45, 70-311 Szczecin, POLAND

*e-mail: mirfray@zut.edu.pl

SUPPORTING INFORMATION

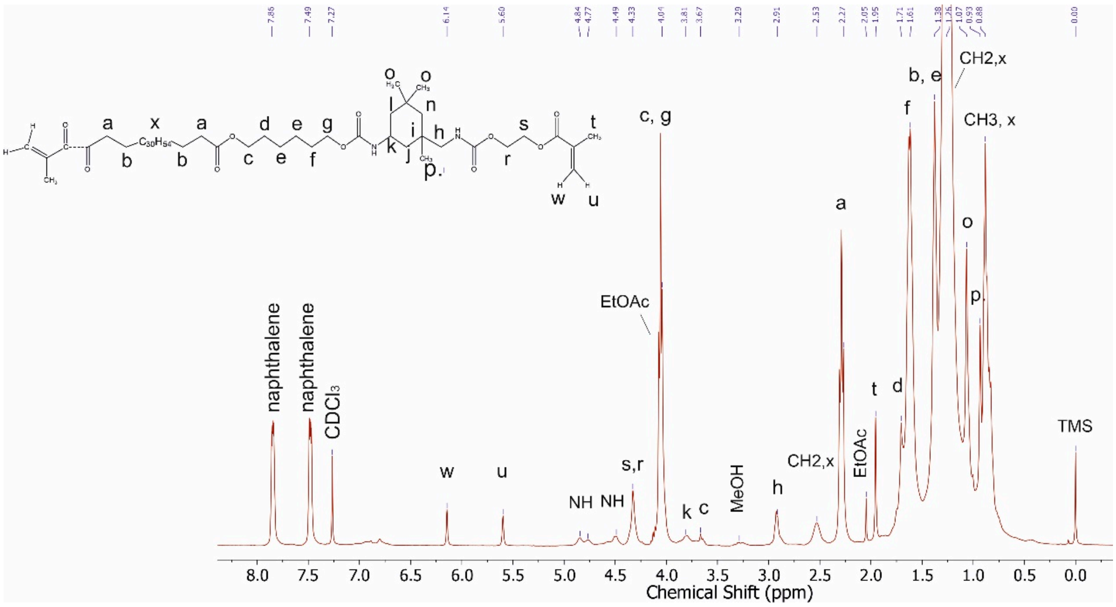

Figure S1 ^{1}H NMR of ester-urethane macromonomer

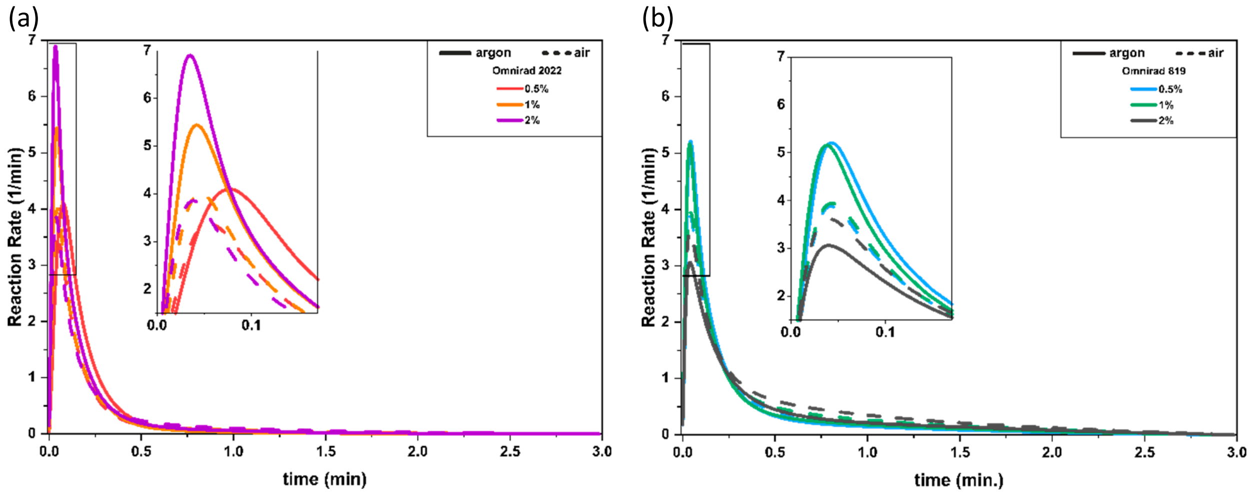

Figure S2 Reaction rate as a function of time for different concentrations 0.5, 1, and 2% wt. of photoinitiator Omnirad 2022 (a) and Omnirad 819 (b) at 20 mW/cm^2

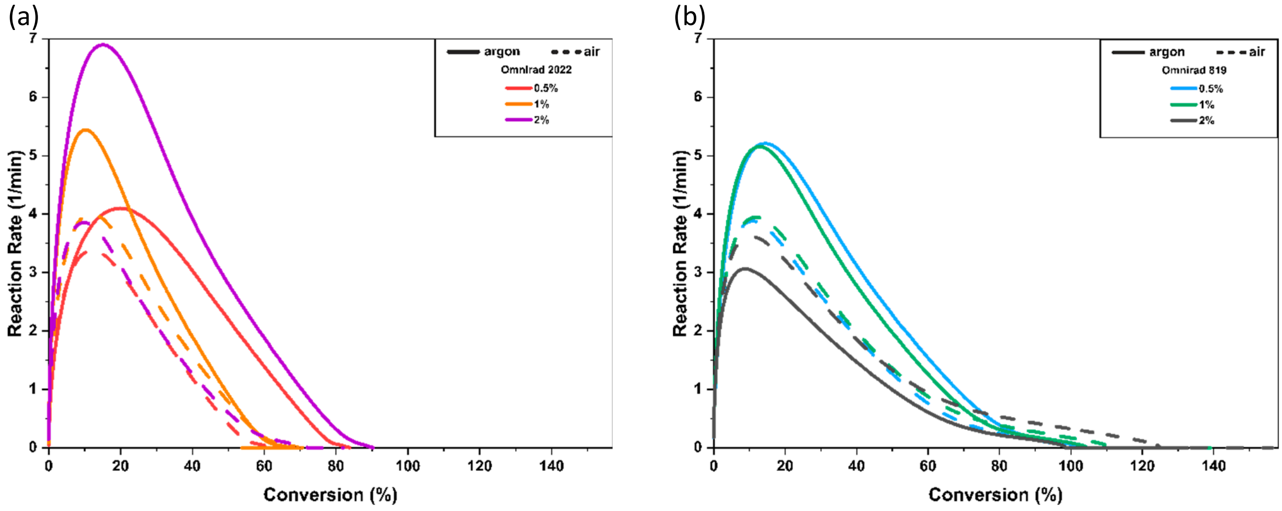

Figure S3 Reaction rate as a function of conversion for different concentrations 0.5, 1, and 2% wt. of photoinitiator Omnirad 2022 (a) and 819 (b) at 20 mW/cm^2

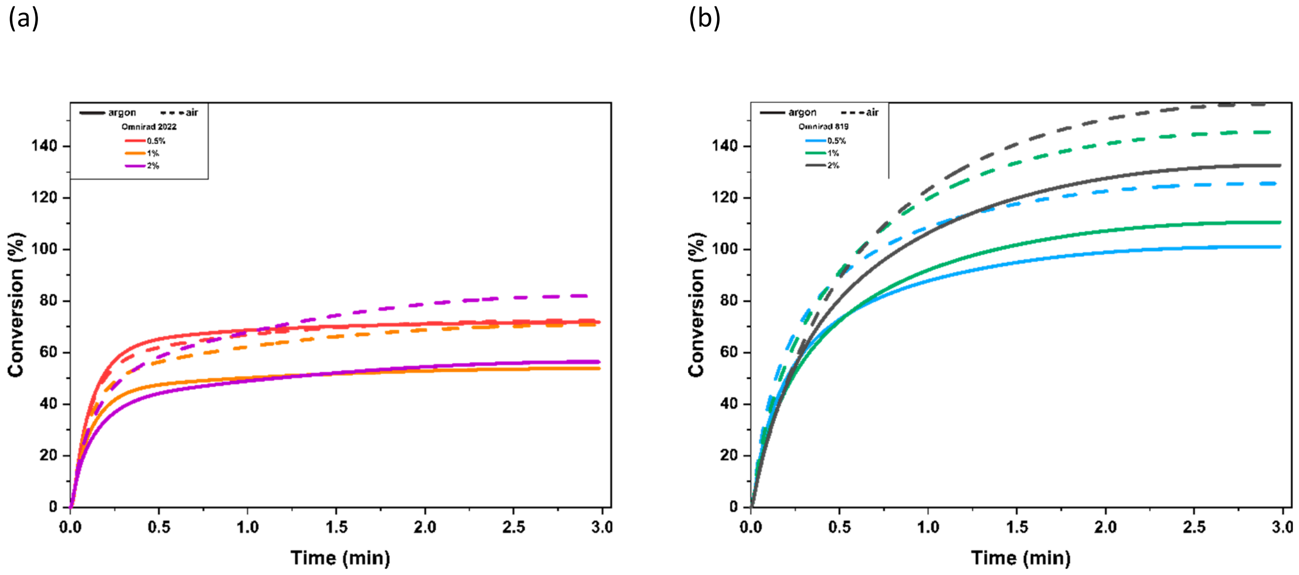

Figure S4 Monomer conversion as a function of time for different concentrations 0.5, 1 and 2% wt. of photoinitiator Omnirad 2022 (a) and 819 (b) at 50mW/cm²

(a) (b)

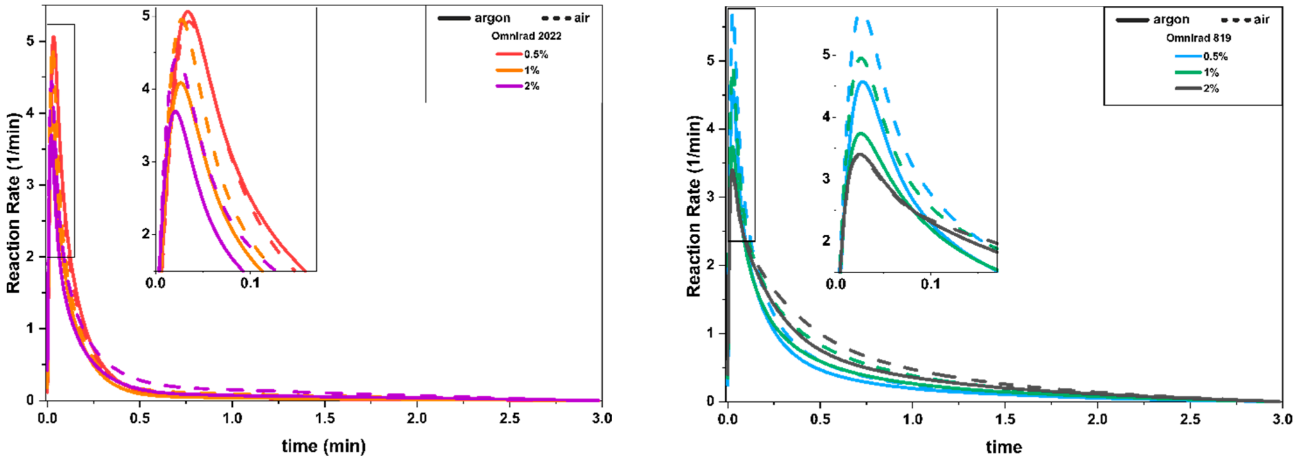

Figure S5 Reaction rate as a function of time for different concentrations 0.5, 1 and 2% wt. of photoinitiator Omnirad 2022 (a) and 819 (b) at 50 mW/cm^2

(a) (b)

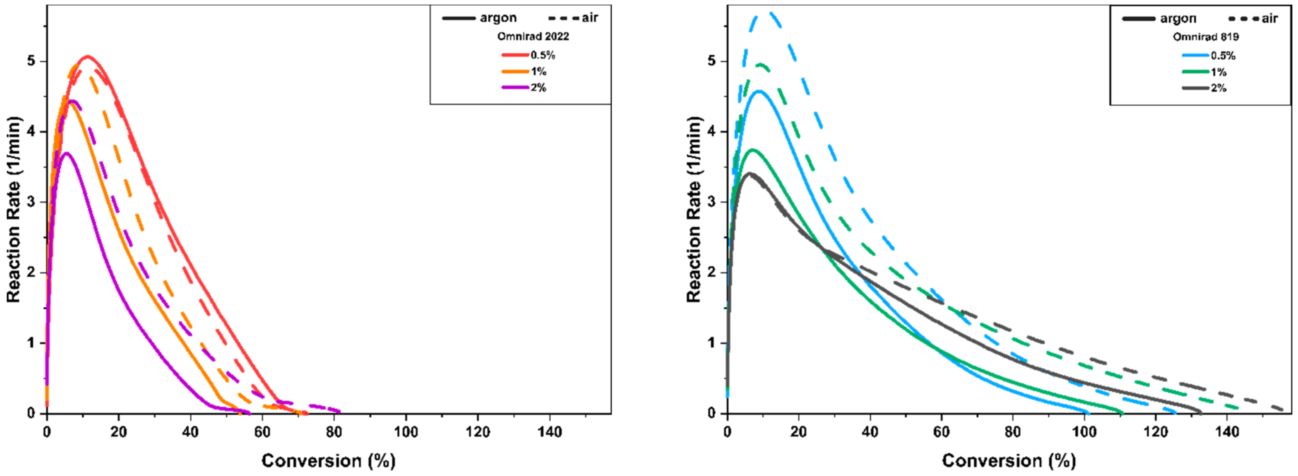

Figure S6 Reaction rate as a function of conversion for different concentrations 0.5, 1, and 2% wt. of photoinitiator Omnirad 2022 (a) and 819 (b) at 50 mW/cm^2

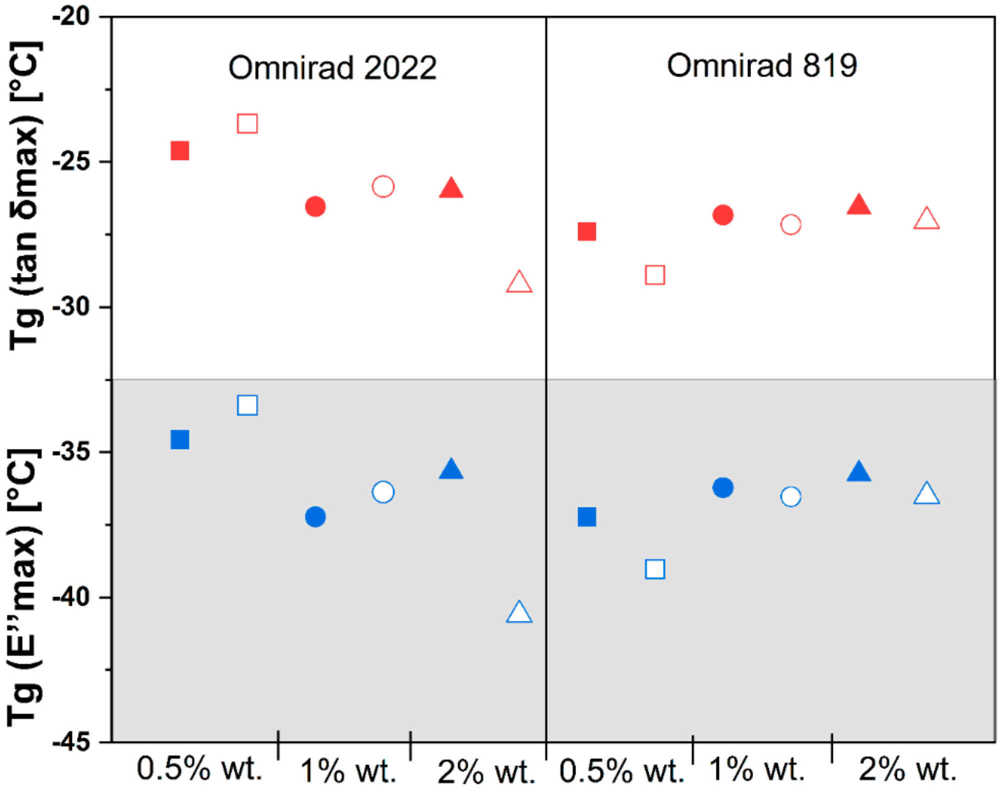

Figure S7 Glass transition temperature of samples cured in argon (closed symbols) and air atmospheres (open symbols) with the use of 20 mW/cm^2 light intensity.

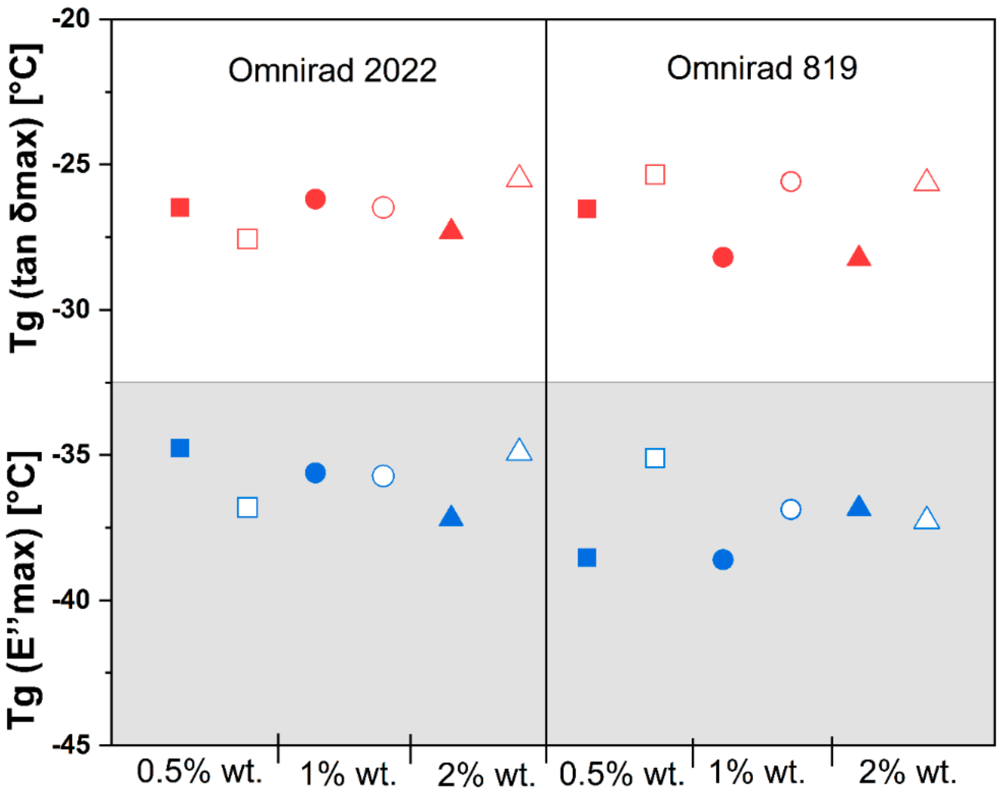

Figure S8 Glass transition temperature of samples cured in argon (closed symbols) and air atmospheres (open symbols) with the use of 50 mW/cm² light intensity.

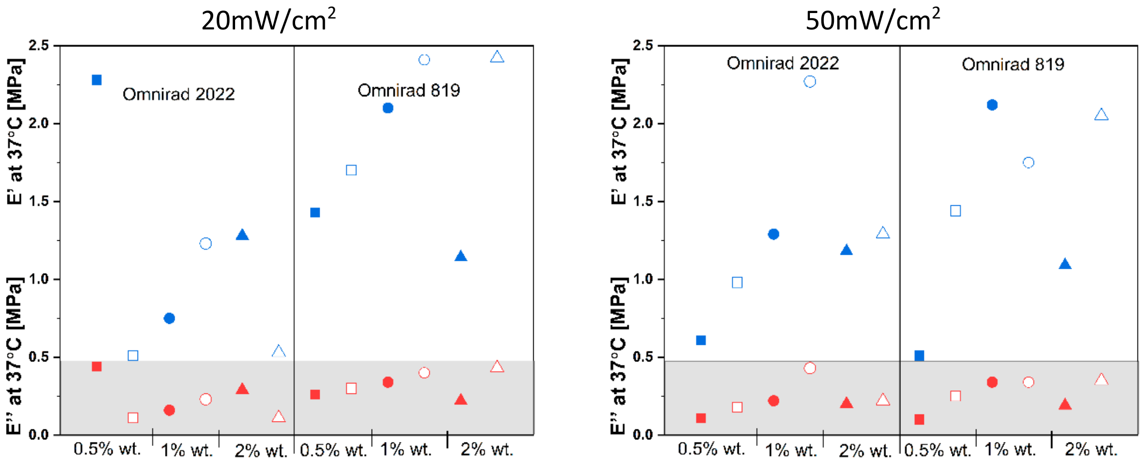

Figure S9 Storage (blue) and loss (red) modulus at 37°C of samples cured in argon (closed symbols) and air atmospheres (open symbols)

Table S1 Parameters of the photopolymerization process at a light intensity of 20 mW/cm²

photoinitiator	Concentration of photoinititor [%wt.]	atmosphere	$t_{1/2}$[min]	Entalpia [J/g]	C_{max} [%]	R_{max} [1/min]	C_u [%]	T_{max}
Omnirad 2022	0.5	Argon	0.14±0.01	17.68±0.49	81.9±2.3	4.46±0.35	17.72±1.84	37.7±0.0
		Air	0.58±0.05	13.49±0.01	62.5±0.1	3.14±0.32	11.35±0.98	37.6±0.1
	1	Argon	0.12±0.01	16.82±0.19	77.8±0.9	5.18±0.38	13.78±0.47	37.7±0.1
		Air	0.27±0.08	15.48±0.68	71.7±3.1	4.39±0.54	12.06±0.05	37.7±0.1
	2	Argon	0.10±0.02	18.65±1.20	86.3±5.6	6.17±1.03	14.71±0.58	37.8±0.1
		Air	0.28±0.11	16.61±1.51	76.9±7.0	4.36±0.72	10.63±1.19	37.7±0.0
Omnirad 819	0.5	Argon	0.16±0.01	22.90±0.93	106.0±4.3	5.13±0.11	13.93±0.88	37.8±0.0
		Air	0.22±0.02	21.78±0.62	100.9±2.9	4.19±0.43	11.05±0.71	37.7±0.1
	1	Argon	0.21±0.06	23.17±0.87	107.3±4.0	4.65±0.72	11.53±2.00	37.8±0.0
		Air	0.20±0.04	25.08±1.52	116.1±7.0	4.44±0.70	11.48±0.36	37.7±0.0
	2	Argon	0.25±0.06	20.63±2.54	95.5±11.8	3.67±0.86	9.64±1.32	37.6±0.1
		Air	0.21±0.02	26.90±0.14	124.5±0.6	3.66±0.06	11.11±1.26	37.7±0.0

Table S2 Kinetic parameters of the Sestak-Berggren model for photocrosslinking at a light intensity of 20 mW/cm²

photoinitiator	Concentration of photoinitiator [%wt.]	atmosphere	k	n	m
Omnirad 2022	0.5	Argon	0.54±0.07	3.39±0.25	0.74±0.03
		Air	0.68±0.21	6.12±0.24	0.80±0.08
	1	Argon	0.59±0.03	3.80±0.07	0.67±0.01
		Air	0.60±0.01	4.85±0.38	0.68±0.02
	2	Argon	0.46±0.02	3.03±0.45	0.54±0.06
		Air	0.38±0.01	4.32±0.68	0.53±0.02
Omnirad 819	0.5	Argon	0.26±0.00	2.43±0.20	0.42±0.00
		Air	0.19±0.02	2.55±0.06	0.36±0.01
	1	Argon	0.22±0.03	2.73±0.36	0.38±0.02
		Air	0.16±0.01	2.11±0.24	0.29±0.02
	2	Argon	0.15±0.05	2.42±0.09	0.30±0.05
		Air	0.11±0.01	1.73±0.02	0.23±0.03

Table S3 Parameters of the photopolymerization process at a light intensity of 50 mW/cm^2

photoinitiator	Concentration of photoinititor [%wt.]	atmosphere	$t_{1/2}$[min]	Entalpia [J/g]	C_{max} [%]	R_{max} [1/min]	C_u [%]	T_{max}
Omnirad 2022	0.5	Argon	0.24±0.07	15.01±0.67	69.5±3,1	4.84±0.31	10.51±0.59	38.0±0.0
		Air	0.38±0.18	14.84±1.14	68.7±5.3	4.64±0.4	10.2±1.45	38.0±0.0
	1	Argon	2.06±1.15	10.95±0.05	50.7±4.4	3.91±0.26	7.01±0.46	37.9±0.0
		Air	0.38±0.01	15.15±0.15	70.2±0.7	5.03±0.11	8.5±0.03	38±0.0
	2	Argon	1.26±0.01	12.11±0.10	56.1±0.4	3.67±0.03	5.9±0.24	37.5±0.7
		Air	0.41±0.09	16.63±1.53	77.0±7.1	4.4±0.05	6.94±0.5	37.9±0.0
Omnirad 819	0.5	Argon	0.23±0.03	21.8±0.05	100.9±0.024	4.36±0.30	8.2±0.98	37.9±0.0
		Air	0.18±0.04	25.17±2.75	116.5±12.75	5.20±0.74	9.2±1.52	38.00±0.0
	1	Argon	0.24±0.01	24.80±1.30	114.8±6.0	4.00±0.36	6.9±0.53	38.0±0.0
		Air	0.14±0.04	33.89±3.45	156.9±16.0	6.14±1.68	10.4±1.65	38.1±0.1
	2	Argon	0.25±0.04	28.34±0.43	131.2±2.0	3.23±0.25	6.5±0.17	38.0±0.0
		Air	0.02±0.01	35.76±2.77	165.6±12.9	3.75±0.52	7.0±0.92	38.1±0.0

Table S4 Kinetic parameters of the Sestak-Berggren model for photocrosslinking at a light intensity of 50 mW/cm²

photoinitiator	Concentration of photoinitiator [%wt.]	atmosphere	k	n	m
Omnirad 2022	0.5	Argon	0.63±0.03	4.99±0.45	0.65±0.02
		Air	0.62±0.02	5.55±0.86	64±0.01
	1	Argon	0.84±0.14	8.77±1.88	0.70±0.05
		Air	0.52±0.01	5.59±0.08	0.54±0.00
	2	Argon	0.36±0.04	7.41±0.06	0.47±0.04
		Air	0.23±0.04	4.65±0.72	0.34±0.03
Omnirad 819	0.5	Argon	0.12±0.02	2.29±0.04	0.21±0.03
		Air	0.13±0.02	1.81±0.34	0.18±0.01
	1	Argon	0.07±0.01	1.52±0.23	0.10±0.05
		Air	0.11±0.04	1.05±0.02	0.11±0.03
	2	Argon	0.05±0.01	0.94±0.01	0.5±0.03
		Air	0.05±0.00	0.49±0.04	0.02±0.01

Table S5 DMTA results

Light intensiti [mW/cm²]	Photoinitiator	Concentration of photoinitiator [%wt.]	Curing atmosphere	Tg (E''_{max}) [°C]	Tg (tan δ_{max}) [°C]	E' at 37 °C [MPa]	E'' at 37°C [MPa]	Tan δ at 37 °C
20	Omnirad 2022	0.5	Air	-33.38	-23.69	0.51	0.11	0.22
			Argon	-34.56	-24.61	2.28	0.44	0.19
		1	Air	-36.37	-25.85	1.23	0.23	0.19
			Argon	-37.22	-26.54	0.75	0.16	0.21
		2	Air	-40.60	-29.23	0.53	0.11	0.21
			Argon	-35.66	-25.98	1.28	0.29	0.22
	Omnirad 819	0.5	Air	-39.02	-28.89	1.70	0.30	0.18
			Argon	-37.22	27.40	1.43	0.26	0.18
		1	Air	-36.53	-27.16	2.41	0.40	0.17
			Argon	-36.22	-26.83	2.10	0.34	0.16
		2	Air	-36.50	-27.04	2.42	0.43	0.18
			Argon	-35.75	-26.55	1.14	0.22	0.19
50	Omnirad 2022	0.5	Air	-36.80	-27.56	0.98	0.18	0.18
			Argon	-34.76	-26.48	0.61	0.11	0.19

		1	Air	-35.72	-26.48	2.27	0.43	0.19
			Argon	-35.61	-26.19	1.29	0.22	0.18
		2	Air	-34.92	-25.51	1.29	0.22	0.21
			Argon	-37.19	-27.32	1.18	0.20	0.17
	Omnirad 819	0.5	Air	-35.10	-25.35	1.44	0.25	0.17
			Argon	-38.53	-26.53	0.51	0.10	0.19
		1	Air	-36.87	-25.59	1.75	0.34	0.20
			Argon	-38.60	-28.19	2.12	0.34	0.16
		2	Air	-37.27	-25.65	2.05	0.35	0.17
			Argon	-36.84	-28.24	1.09	0.19	0.17
